# Beyond the Colon: *Clostridioides difficile* Enteritis Presenting Following a Nissen Fundoplication

**DOI:** 10.14309/crj.0000000000002081

**Published:** 2026-04-09

**Authors:** Fnu Alvina, Dayana Nasr, Kelita Singh

**Affiliations:** 1 Department of Internal Medicine, SUNY Upstate Medical University, Syracuse, NY; 2 Division of Gastroenterology, SUNY Upstate Medical University, Syracuse, NY; 3 Division of Gastroenterology, SUNY Upstate Medical University, Syracuse, NY

**Keywords:** *Clostridioides difficile* enteritis, *Clostridioides difficile* infection, small bowel, Nissen fundoplication, postoperative sepsis

## Abstract

*Clostridioides difficile* enteritis (CDE) is a rare condition usually seen in patients with recent abdominal surgery and/or antibiotics. A 49-year-old male patient presented following a Nissen fundoplication surgery with watery diarrhea, abdominal pain, tenderness, tachycardia, and hypotension. Stool polymerase chain reaction for *C. difficile* was positive, enzyme immunoassay negative. Abdominal CT was suggestive of enteritis. He was treated with a course of oral vancomycin. This study highlights that CDE can occur following other surgeries apart from ileostomy/colectomy and should be on the differential for high-risk patients (hospitalization, proton pump inhibitor (PPI) use, and antibiotic exposure).

## INTRODUCTION

*Clostridioides difficile* infection (CDI) is a major contributor to healthcare-associated infections worldwide.^1^ The clinical presentation is highly variable from mild diarrhea to fulminant colitis and toxic megacolon. In severe cases, CDI can lead to bowel perforation.^2^ CDI typically involves the colon; however, it can affect the small bowel in rare cases, causing *Clostridioides difficile* enteritis (CDE).

CDE predominantly occurs following colectomy with an incidence of 5.1% usually within the first year^3^ and is associated with mortality rates of 23%. However, it can also occur in patients with an intact colon particularly in the context of recent hospitalization, antibiotic exposure, and gastric acid suppression.^4^

Here, we present a case of CDE following an elective hiatal hernia repair with a Nissen fundoplication.

## CASE REPORT

A 49-year-old male patient with a history of hiatal hernia status post repair with Nissen fundoplication presented 2 weeks postoperatively with severe generalized abdominal pain, bloating, and multiple episodes of nonbloody, watery diarrhea (4–6 bowel movements a day). There was a concern for small bowel obstruction on abdominal computed tomography (CT) thought to be a postoperative ileus, for which he was treated conservatively and discharged home.

He returned to the hospital 3 days later with worsening symptoms of generalized abdominal pain and multiple episodes (>8 bowel movements/day) of nonbloody, watery diarrhea. He was found to be hypotensive up to 80/40 mm Hg, tachycardic 110, and afebrile. Physical examination revealed diffuse abdominal tenderness without peritoneal signs.

**Figure 1.**
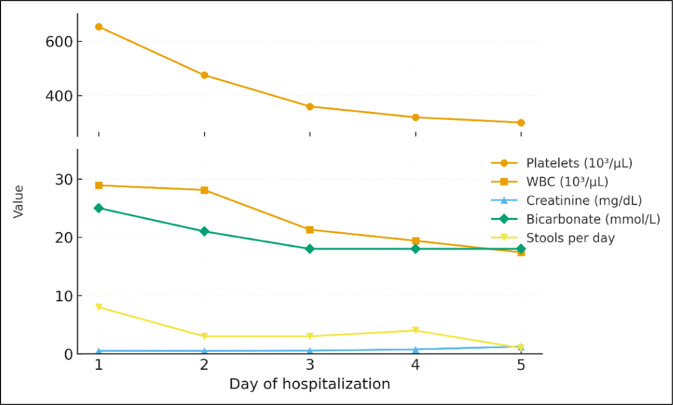
Graph demonstrating the laboratory values and number of bowel movements during hospitalization (normal range—platelets: 150–450 × 10^3^/µL; WBC: 4–11 × 10^3^/µL; creatinine: ∼0.6–1.3 mg/dL; bicarbonate: 22–29 mmol/L). WBC, white blood cell.

Laboratory values demonstrated leukocytosis 28.9 × 10^3^, hemoglobin 18 g/dL, platelets 653 × 10^3^, creatinine 1.26 mg/dL, bicarbonate 18 mmol/L, potassium 4.4 mmol/L, and lactic acid 1 mmol/L, on admission (Figure 1).

A stool polymerase chain reaction panel showed *C. difficile* polymerase chain reaction positive, enzyme immunoassay negative. He had no history of CDI. We interpreted this result in the setting of his overall clinical presentation of diarrhea, abdominal pain, recent history of hospitalization, and laboratory findings of leukocytosis.

**Figure 2.**
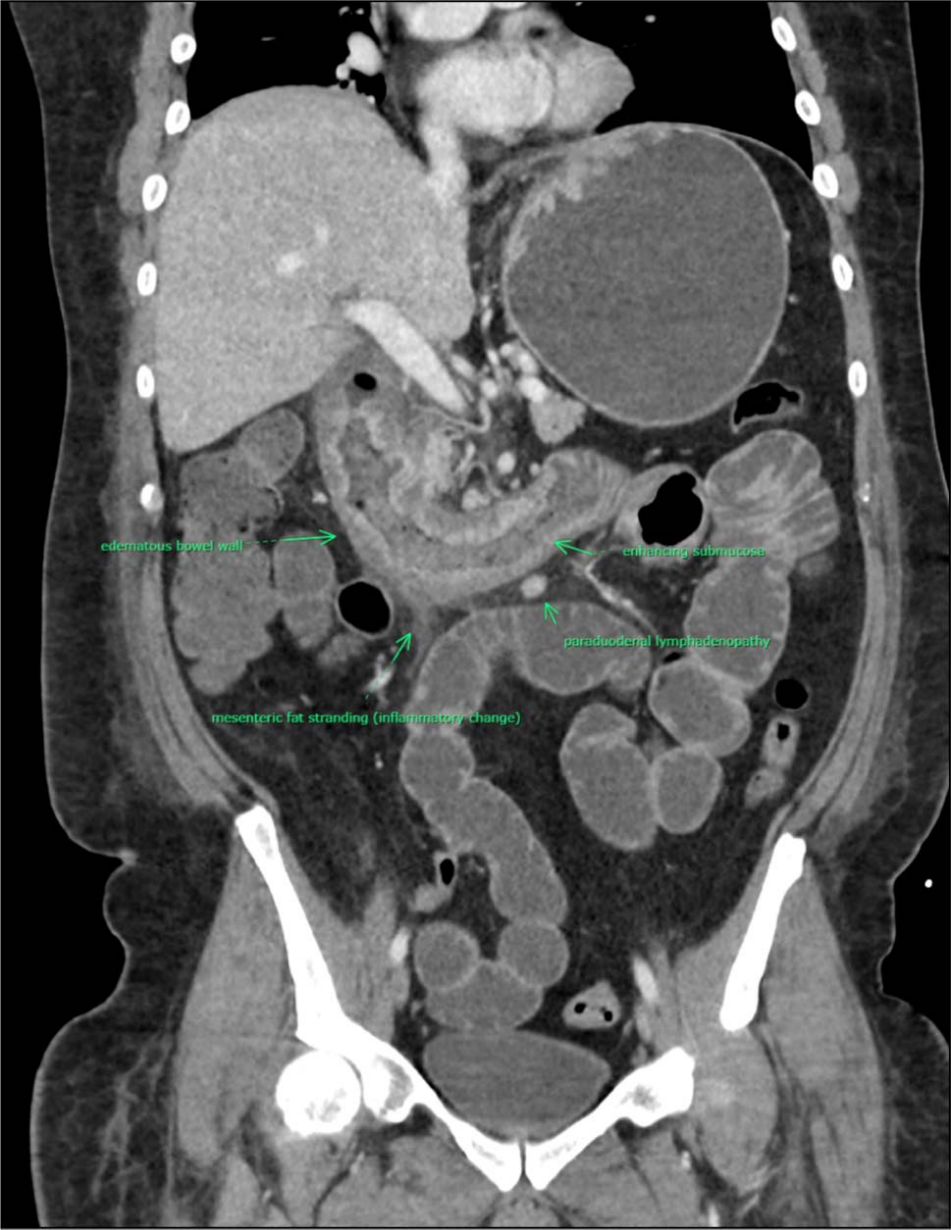
Abdominal and pelvic CT scan suggestive of small bowel enteritis.

In addition, a CT scan showed evidence of loculated fluid collection adjacent to the distal esophagus thought to be a postsurgical fluid collection, and thickening of the proximal jejunum and fluid-filled ileum suggestive of enteritis. On CT imaging, there was no evidence of colitis (Figure 2).

The patient's presentation was therefore most consistent with CDE. The patient was treated with oral vancomycin 125 mg 4 times daily to complete a 10-day course, following which his symptoms improved. Adjunctive metronidazole therapy was not added as there was no concern for fulminant *C. difficile* infection, given the absence of ileus, toxic megacolon, or significant metabolic derangements in addition to the rapid response to oral vancomycin.

## DISCUSSION

CDE remains a rare diagnosis with around 50–80 cases reported worldwide.^5^ Prior antibiotic use is known to alter the commensal flora allowing *C. difficile* to proliferate. Another known predisposing factor is gastric acid suppression through H2-blockers and proton pump inhibitor (PPI) use that act through the same mechanism.^4^ Toxigenic strains then produce toxins A and B, leading to mucosal injury, increased secretion, and inflammation. In CDE, this damage is confined predominantly to the small bowel, causing high-volume diarrhea, abdominal pain, and sepsis.^6^ Most published cases of CDE occur following lower gastrointestinal surgeries such as total colectomy, creation of an ileostomy, or ileal pouch-anal anastomosis. It is also more frequently seen in patients with Inflammatory bowel disease.^7^ In their systematic review of 83 cases in 2013, Dineen et al^4^ noted that the majority of CDE occurred in patients with recent antibiotic therapy and/or abdominal surgery.

Our patient was on a proton pump inhibitor chronically and was treated with cefazolin 2 g once in the perioperative period. However, he did not have a history of inflammatory bowel disease or prior lower gastrointestinal surgeries. Unlike the majority of published CDE cases, the colon remained intact, underscoring that substantial colonic involvement or resection is not required for *C. difficile* to involve the small bowel. This case demonstrates that CDE should be considered across a broader spectrum of postoperative settings.

The diagnosis of CDE is clinically challenging as it can mimic the presentation of postoperative complications such as small bowel obstruction and ileus as seen in our patient. Imaging can often be nonspecific and may show diffuse small bowel wall thickening, fluid filled loops, and intramural air, amongst other findings.^6^ Owing to the nonspecific presentation, diagnosis relies on a high degree of clinical suspicion and microbiological testing. The current guidelines were developed by a multidisciplinary panel representing the Infectious Diseases Society of America and the Society for Healthcare Epidemiology of America, using stool toxin enzyme immunoassays and/or nucleic acid amplification tests to detect toxigenic *C. difficile* in patients with compatible symptoms.^8^

The treatment of CDE parallels that of colonic CDI. According to Infectious Diseases Society of America/Society for Healthcare Epidemiology of America guidelines, the first-line therapy for nonfulminant disease consists of oral vancomycin or fidaxomicin, with intravenous metronidazole reserved for severe or fulminant presentations.^8^

In summary, this case highlights CDE as a potential, albeit rare, cause of postoperative sepsis following hospitalization for the Nissen fundoplication procedure. While prior reports show that bowel surgeries such as colectomy or ileostomy confer a risk of CDE, this case highlights that CDE can also arise after other types of surgeries in conjunction with risk factors such as hospitalization, PPI use, and antibiotic exposure.

Early diagnosis, supportive care including aggressive fluid resuscitation, nutritional support, electrolyte replacement, and appropriate antibiotic therapy are integral to preventing complications requiring surgical intervention. As such, CDE should be on the differential diagnosis for patients with the risk factors as demonstrated by this case so that treatment can be initiated early.

## DISCLOSURES

Author contributions: Conception/design—data acquisition, analysis, or interpretation of data for the work: F. Alvina, D. Nasr, K. Singh. Drafting/revising—drafting the manuscript or revising it critically for important intellectual content: F. Alvina, D. Nasr, K. Singh. Final approval: providing final approval of the version to be published: F. Alvina, D. Nasr, K. Singh. Accountability—agreeing to be accountable for all aspects of the work, ensuring accuracy and integrity: F. Alvina, D. Nasr, K. Singh. F. Alvina is the article guarantor.

Financial disclosure: None to report.

Informed consent was obtained for this case report.

Disclosures: This research received no specific grant from any funding agency in the public, commercial, or not-for-profit sectors and there are no conflicts of interest. There has been no concurrent submission to another journal. There was no prior presentation of the case report at a professional meeting. Generative artificial intelligence was not used in the article.

## ABBREVIATIONS:

CDE: *Clostridioides difficile* enteritis
CDI: *Clostridioides difficile* infection
IBD: Inflammatory Bowel Disease
PPI: Proton pump inhibitor
